# Increasing Brain Protein O-GlcNAc-ylation Mitigates Breathing Defects and Mortality of Tau.P301L Mice

**DOI:** 10.1371/journal.pone.0084442

**Published:** 2013-12-23

**Authors:** Peter Borghgraef, Clément Menuet, Clara Theunis, Justin V. Louis, Herman Devijver, Hervé Maurin, Caroline Smet-Nocca, Guy Lippens, Gerard Hilaire, Harrie Gijsen, Dieder Moechars, Fred Van Leuven

**Affiliations:** 1 Experimental Genetics Group - LEGTEGG, KULeuven, Leuven, Belgium; 2 MP3-Respiration, UMR CNRS 6231, Faculté Saint-Jérôme, Marseille, France; 3 Groupe RMN-Glycobiologie, CNRS, University de Lille, Villeneuve d'Ascq, France; 4 Department Neuroscience, Janssen Research & Development, Beerse, Belgium; Boston University School of Medicine, United States of America

## Abstract

The microtubule associated protein tau causes primary and secondary tauopathies by unknown molecular mechanisms. Post-translational O-GlcNAc-ylation of brain proteins was demonstrated here to be beneficial for Tau.P301L mice by pharmacological inhibition of O-GlcNAc-ase. Chronic treatment of ageing Tau.P301L mice mitigated their loss in body-weight and improved their motor deficits, while the survival was 3-fold higher at the pre-fixed study endpoint at age 9.5 months. Moreover, O-GlcNAc-ase inhibition significantly improved the breathing parameters of Tau.P301L mice, which underpinned pharmacologically the close correlation of mortality and upper-airway defects. O-GlcNAc-ylation of brain proteins increased rapidly and stably by systemic inhibition of O-GlcNAc-ase. Conversely, biochemical evidence for protein Tau.P301L to become O-GlcNAc-ylated was not obtained, nor was its phosphorylation consistently or markedly affected. We conclude that increasing O-GlcNAc-ylation of brain proteins improved the clinical condition and prolonged the survival of ageing Tau.P301L mice, but not by direct biochemical action on protein tau. The pharmacological effect is proposed to be located downstream in the pathological cascade initiated by protein Tau.P301L, opening novel venues for our understanding, and eventually treating the neurodegeneration mediated by protein tau.

## Introduction

Conjugation of β-N-acetylglucosamine to Ser/Thr residues is a reversible post-translational modification of many proteins, controlled by two unique enzymes: O-GlcNAc transferase (OGT) and β-N-acetyl-glucosaminidase (OGA) [1-5]. Hundreds of proteins become O-GlcNAc-ylated in all cells and tissues, but many questions remain about the specificity, the structural and functional significance, and the physiological and pathological repercussions in vivo. The important physiological importance is surmised from the perinatal lethality of mice with inactivated X-linked OGT genes [6,7]. Because implications of O-GlcNAc-ylation in peripheral organs and systems is well reviewed [1-7], we focus here on CNS.

Both OGT and OGA are abundant in brain, particularly in the hippocampus, making O-GlcNAc-ylation subject to speculation for important roles in health and disease [8,9]. Moreover, neuron-specific deficiency of OGT caused postnatal lethality and motor defects [6,7] supporting the hypothesis of important functions in CNS. The lethal outcome of OGT-based genetic models prevents analysis of roles of OGT in adult and ageing brain, which is our main interest. Pharmacological inhibition of OGT is hampered, if not prohibited, by its structural complexity [2,3] while effective inhibitors are available for OGA, the enzyme that removes O-GlcNAc moieties from proteins [2,3]. A promising inhibitor denoted Thiamet-G [10,11] remains to be independently tested and validated in dedicated disease models in vivo.

The only known physiological function of protein tau is binding to microtubules. In adult brain this is controlled by variable and complex phosphorylation of protein tau, which eventually also mediates its pathological aggregation causing tauopathies [12-15]. The large number of Ser/Thr residues in the naturally unfolded protein tau allows for a vast number of phospho-tau isoforms, still beyond detailed experimental analysis. Moreover, kinases traditionally known as tau-kinases, e.g. GSK-3 kinases and cdk5, but also MARK/Par1 and others, serve widely different clients in many different signaling pathways, obscuring their contribution to protein tau and tauopathies. Conversely, the interest in protein tau in neuropathology is high, in first instance as direct cause of many primary tauopathies, but even more by its obligatory co-morbidity in Alzheimer's disease (AD) [12-15]. 

O-GlcNAc-ylation has been proposed to compete with or counteract phosphorylation of different neuronal cell-surface, nuclear and cytoplasmic proteins [1-5]. While protein tau can be O-GlcNAc-ylated in vitro in recombinant systems and in some transfected cell-lines in culture [16-21], the data remain circumstantial because conclusive biochemical evidence is lacking in vivo, in mouse and human brain. 

The designed OGA inhibitor denoted Thiamet-G [10] was tested here for the first time independently in vivo, in acute and chronic studies in Tau.P301L mice as validated pre-clinical model for tauopathy [22-26]. The compound increased the biochemical level of a large number of O-GlcNAc-ylated brain proteins rapidly (hours) and stably (months) in wild-type and transgenic mouse brain, proving that it entered CNS, while long-term administration revealed no major negative drawbacks. On the contrary, inhibition of OGA mitigated the reduction in body-weight and the motor deficits, when administered per os over a period of 10 weeks to ageing Tau.P301L mice. Significantly more compound-treated than placebo-treated Tau.P301L mice survived, correlating with improved upper airway breathing defects, which we have identified as the prime problem of ageing Tau.P301L mice [22-26]. 

### Materials and MethodsEthics Statement

All mice were maintained in the university central animal house (KULeuven, Leuven, Belgium). All animal experiments were approved by the KULeuven ethical commission and performed according institutional, Belgian and European guidelines (86/609/EEC, 2003/65/EC European Council Directives).

### Mice

Female Tau.P301L transgenic mice in the FVB/N genetic background were used throughout. These mice express the longest human tau isoform with the P301L mutation (tau-4R/2N-P301L) under the mouse *thy1* gene promoter and are homozygous for the transgene [22,24]. Mice deficient in protein tau [27] were obtained from The Jackson Laboratory (Bar Harbor, ME) from stock #004779, *Mapt* ^*tm1*(*EGFP*^)*Klt*/*J*. All transgenic mice were genotyped by standard PCR methods on DNA extracted from tail biopsies.

### Synthesis of OGA inhibitor Thiamet G

Synthesis of 1,2-dideoxy-2'-ethylamino-α-D-glucopyranoso-[2,1-d]-delta-2'-thiazoline (CAS 1009816-48-1) was essentially as reported [10] with minor modification. A solution of 3,4,6-tri-*O*-acetyl-1,2-dideoxy-2'-ethylamino-α-D-glucopyranoso-[2,1-d]-delta-2'-thiazoline in methanol was treated with Amberlite IRA-400(OH-) resin and the mixture was shaken at room temperature for 6 hr. After filtration, and solvent evaporation under reduced pressure, the residue was triturated with hot isopropanol and slowly cooled to room temperature under dropwise addition of heptane. The precipitate was collected by filtration, washed with heptane and dried to yield Thiamet G: C_9_H_16_N_2_O_4_S; mp 170 °C; elemental analysis calculated/experimental: C 43.54/ 43.55, H 6.50/6.44, N 11.28/11.37; confirmed by spectroscopy [10].

### Animal treatment and tissue processing

Thiamet G was dissolved in saline for intraperitoneal injection and for gavage, or in drinking water for administration per os. For brain analysis, mice were euthanized under deep anesthesia (Nembutal) by transcardiac perfusion with ice-cold saline (2 ml/min for 2 min). One hemisphere with half the brainstem was snap-frozen in liquid nitrogen for biochemical analysis. The other hemisphere and half the brainstem was fixed overnight in 4% paraformaldehyde in PBS at 4°C, and then stored in 0.1% sodium azide in PBS at 4°C until sectioning and analysis by immunohistochemistry. 

### Clinical read-outs, motor tests, plethysmography

Mice were monitored daily for body-weight, clasping and general condition. Clasping was scored by lifting mice by the tail: normal score 0, all limbs extended, score 1-4, one to four limbs retracted or apposed to the body. Clasping score 5 was included operationally to define the terminal stage of Tau.P301L mice by the combination of body-weight at or less than 16 gram and maximal clasping-score 4, as explained in the results section. 

The standard automated rotarod tested 5 mice simultaneously on the revolving rod. Mice were trained in 3 sessions each of 3 min at 20 rpm on three consecutive days in the week before pharmacological treatment started. Mice were subsequently tested every 2 weeks on the rod accelerating from 4 to 40 rpm over 300 sec. The time that the mice remained on the rod was recorded automatically. 

Double-chamber plethysmography was performed as described, with mice habituated to the plethysmography procedure before recordings [25,26)]. In brief, we simultaneously measured following parameters: respiratory frequency (Rf, expressed as cycles per min) in conscious, unrestrained mice; chest respiratory movement in the body chamber (chest spirogram, CSp); the resulting airflow in the head respiratory chamber (airflow spirogram, ASp). The ASp/CSp ratio less than unity in Tau.P301L mice demonstrated the upper airway dysfunction, provoking excessive diaphragm and chest movements in attempting to still produce adequate airflow [25,26]. Eventually, physical exhaustion and asphyxia contribute to precocious mortality of the Tau.P301L mice [22-26, this study]. 

### Western blotting

Forebrain or brainstem were homogenized in 6 volumes of homogenization buffer (with a cocktail of proteinase and phosphatase inhibitors) in a Potter-Elvejhem homogenizer with 10 up-and-down strokes of a teflon pestle rotating at 700 rpm [22-26]. Cell debris were removed by centrifugation (17900xg, 15 min) and the supernatant was diluted in sample buffer for SDS-PAGE on 10% Tris-glycine gels. Western blotting used the ECL system [22-26] with primary antibodies specified (Table 1). Western blots were incubated to prevent non-specific binding with 5% non-fat milk-powder in TBS with 0.1% Tween-20 for most antibodies, but with 5% BSA in TBS, 0.1% Tween-20 for the O-GlcNAc specific antibodies (Table 1). The resulting immune reactions were recorded and analyzed digitally with dedicated instrumentation and software (LAS4000, Image-QuantTL, GE Healthcare, Brussels, Belgium).

**Table 1:** Antibodies used.

**Code**	**Antigen / Epitope**	**Source**	**Use**
CTD110.6	O-GlcNAc - adduct	Covance	IHC, WB
RL2	O-GlcNAc - adduct	Thermo Scientific	IHC, WB
1F5.D6	O-GlcNAc - adduct	Millipore	WB
9D1.E4	O-GlcNAc - adduct	Millipore	WB
18B10C7	O-GlcNAc - adduct	Millipore	WB
6D93	O-GlcNAc - adduct	Santa Cruz	WB
ab3925	S400 Tau O-GlcNAc	*	WB
Total tau / Tau-5	human & mouse tau, phosphorylation independent	BD Pharmingen	WB
Human tau / HT7	human tau, phosphorylation independent	Thermo Scientific	IHC, WB, IP
AD2	tau phosphorylated at S396/S404	Biorad	WB
pS396	tau phosphorylated at S396	Invitrogen	WB
pS404	tau phosphorylated at S404	Sigma	WB
AT180	tau phosphorylated at T231	Innogenetics	WB
12E8	tau phosphorylated at S262/S356	ELAN	WB
AT100	tau phosphorylated at T212/S214	Innogenetics	IHC
GSK3ab	total GSK3a (51 kDa) & GSK3b (47 kDa)	Biosource	WB
GSK3ab - pY279/pY216	phosphorylated Y279-GSK3a & Y216-GSK3b	Biosource	WB
GSK3ab - pS21/pS9	phosphorylated S21-GSK3a & S9-GSK3b	Cell Signaling	WB
GSK3a - pS21	GSK3a phosphorylated at S21	Abcam	WB
GSK3b - pS9	GSK3b phosphorylated at S9	Cell Signaling	WB
CD45 / 30-F11	CD45 / LCA	BD Pharmingen	IHC
GFAP	glial fibrillary acidic protein	Dako	IHC
GAPDH / 2D4A7	used as gel-loading control	Novus	WB
Actin / AC-15	used as gel-loading control	Sigma	WB
PKB	Akt1,2,3	Cell Signaling	WB
PKB pS473 (D9E)	PKB phosphorylated at S473	Cell Signaling	WB
PKB pT308 (244F9)	PKB phosphorylated at T308	Cell Signaling	WB
PP2A C-Y307 (E155)	PP2A catalytic subunit phosphorylated at Y307	Epitomics	WB
PP2A C	catalytic subunit of PP2A	**	WB

(*) Gift from D.J. Vocadlo (Toronto, Canada)

(**) Gift from S.M. Dilworth and V Janssen (KULeuven, Leuven, Belgium)

Synthetic peptides and recombinant protein tau were O-GlcNAc-ylated in vitro with recombinant OGT [10,11,21] and used as positive controls in the biochemical analysis. The vector with cDNA coding for human OGT was a gift from S. Walker (Boston). The antibody ab3925 directed against protein tau O-GlcNAc-ylated at residue S400 [10,11] was a gift from D.J. Vocadlo (Toronto). 

### Immunoprecipitation

Total brain protein lysates were subjected to immuno-precipitation with the HT7 antibody, specific for human protein tau by standard procedures recommended by the manufacturer (Crosslink, Pierce, Brussels, Belgium). The immunoprecipitates were collected by centrifugation, dissolved by boiling in SDS-PAGE sample buffer and analyzed by western blotting on 10 % Tris-glycine gels, as described above. 

### Immunohistochemistry

The general procedures were described [22-26]. In brief, free-floating vibratome sections (40 µm) were rinsed in PBS 0.1% Triton (PBST) and incubated in PBST with 10% fetal calf serum to block non-specific binding. Sections were incubated overnight with primary antibodies (Table 1), followed by washing and incubation with the respective secondary antibodies. Signals were developed by 2-step or 3-step procedures as described [22-26]. Final staining was by incubation with 3,3’-diaminobenzidine, 0.3 % H_2_O_2_ in Tris.HCl (pH 7.6). After mounting with Depex, sections were viewed by light microscopy, and images recorded digitally and analyzed by dedicated software (IM500 Image Manager, Leica, Brussels, Belgium).

### Statistical analysis

The dual-chamber plethysmography data are expressed as mean±standard error of the mean (SEM). Normal distribution was assessed, prior to statistical one-way analysis of variance (ANOVA). ASp/CSp ratios were compared using paired Student *t* test and one-way ANOVA for intergroup differences. Data on the number of AT100 neurons, and on clasping were compared statistically by the Mann Whitney test. Optical density, rotarod data and bodyweight data were analyzed statistically by the unpaired 2 tailed *t* test. Additional details of statistical methods of analysis are specified in the figure legends and text, where appropriate. Statistical analysis was performed by dedicated software (Prism 5, GraphPad, La Jolla, CA). Statistical differences were judged significant at p<0.05.

### Results

### Acute OGA inhibition increased O-GlcNAc-ylation of brain proteins in vivo

In preliminary acute experiments we established that Thiamet-G, administered intraperitoneally or per os, effectively increased O-GlcNAc-ylation of brain proteins in wild-type mice and in Tau.P301L mice. These acute effects were dependent both on time (maximal at 4-8 hrs) and proportional to the dose (up to 500 mg/kg). The compound was confirmed to be stable in solution [10] and was administered in subsequent sub-acute experiments by gavage, and in later chronic experiments dissolved in normal drinking water, supplied in the home cage to reduce stress by excessive handling of old Tau.P301L mice. 

**Figure 1:**
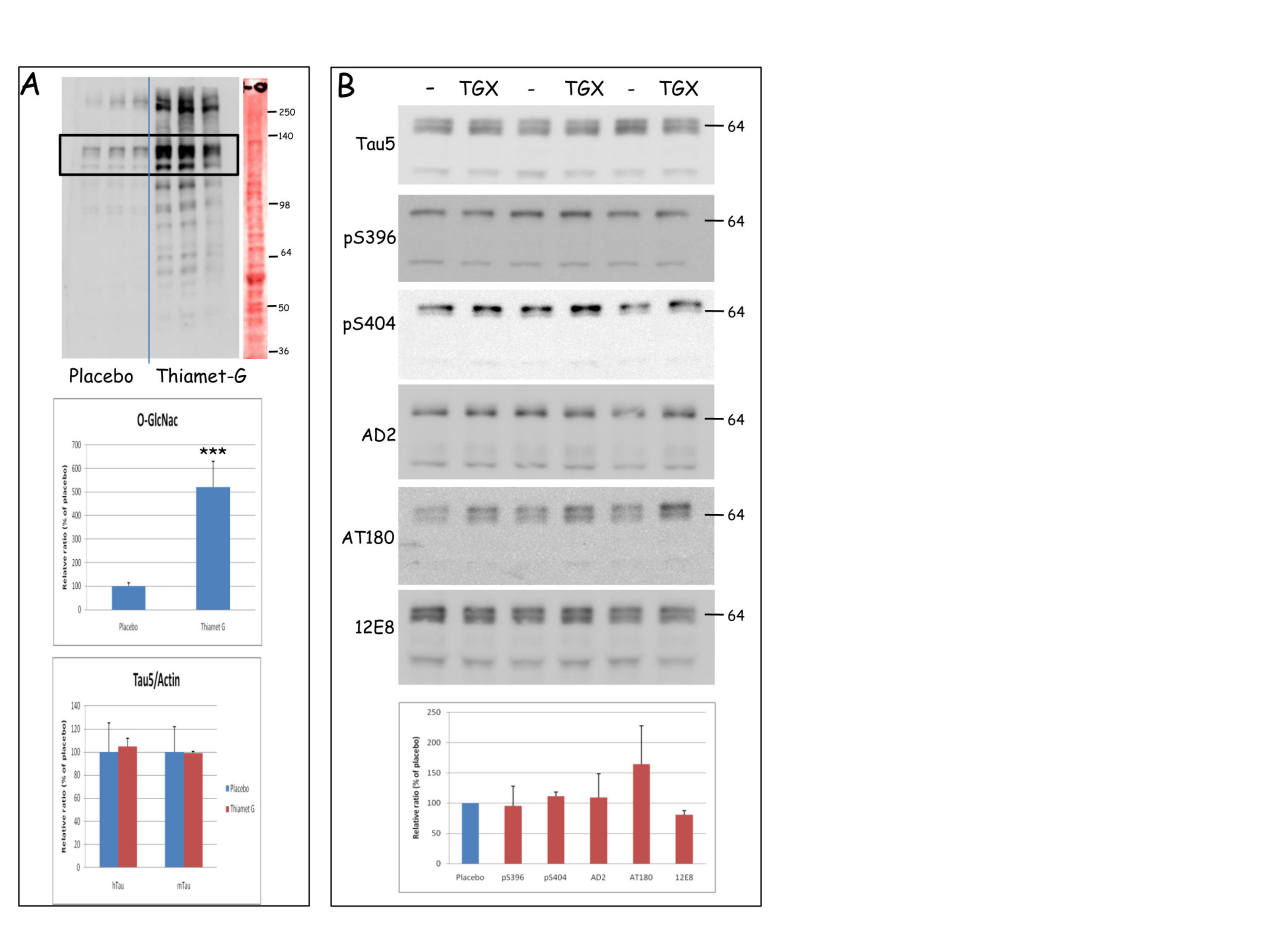
Thiamet-G increased O-GlcNAc-ylation of brain proteins in Tau.P301L mice. A. The upper panel is a representative western blot with antibody CTD110.6 of total forebrain protein extracts from 3 untreated and 3 Thiamet-G treated Tau.P301L mice. The utmost right lane is a representative total protein stain with Ponceau red of one of the extracts. Quantification of the major O-GlcNAc-ylated proteins of unknown identity (120-140 kDa, boxed in the top panel) demonstrated the 5-fold increase in O-GlcNAc-ylation (middle panel) without affecting total levels of human transgenic Tau.P301L protein or mouse endogenous protein tau (denoted hTau and mTau in bottom panel). B. Biochemical analysis by western blotting of total protein tau (Tau5) and its phosphorylated isoforms specified or denoted by the antibody acronym (captions on the left, details in Table 1). Total forebrain homogenates from Tau.P301L mice, untreated (lanes marked "-") or Thiamet-G treated (lanes denoted TGX) analyzed by western blotting with the antibodies indicated. Representative western blots are shown. Bottom panel: quantitative data (mean±SEM) from all mice (placebo n=13, Thiamet-G n=13) .

Biochemically, O-GlcNAc-ylation of many proteins was increased by Thiamet-G in brain of Tau.P301L-mice between 3 to 6-fold for most proteins (Figure 1A, upper and middle panels). Compared to total proteins loaded (Figure 1A, right lane, Ponceau red staining), the selective nature of O-GlcNAc-ylation was evident: not the most abundant brain proteins were O-GlcNAc-ylated, while heavily O-GlcNAc-ylated proteins were not the most abundant. 

Unexpectedly, these initial acute experiments revealed no O-GlcNAc-labeled protein in the brain extracts that corresponded closely in electrophoretic mobility to human Tau.P301L (64-68 kDa) or to mouse protein tau (~55kDa). Total levels of human or mouse protein tau were not affected (Figure 1A, lower panel; Figure 1B). Moreover, most phospho-epitopes of Tau.P301L were not markedly affected by acute Thiamet-G treatment, while reaction with AT180, defining a typical GSK3β-dependent epitope, even tended to be increased (Figure 1B).

### Breathing defects mitigated by sub-acute OGA inhibition in ageing Tau.P301L mice

We went on to defined that the upper airway defects in symptomatic Tau.P301L mice [25,26] were positively affected by OGA inhibition. 

First, we re-established in wild-type mice with the identical FVB/N genetic background as Tau.P301L mice [22] the baseline amplitudes of airflow versus chest spirograms under normocapnia and hypercapnia (2.07±0.27 and 1.93±0.02, respectively), conform our reported data [25,26]. Administration of Thiamet-G in drinking water (2.5 mg/ml) for up to 3 days, increased these values somewhat in wild-type mice (2.58±0.14 and 2.52±0.29, respectively in normocapnia and hypercapnia). 

**Figure 2:**
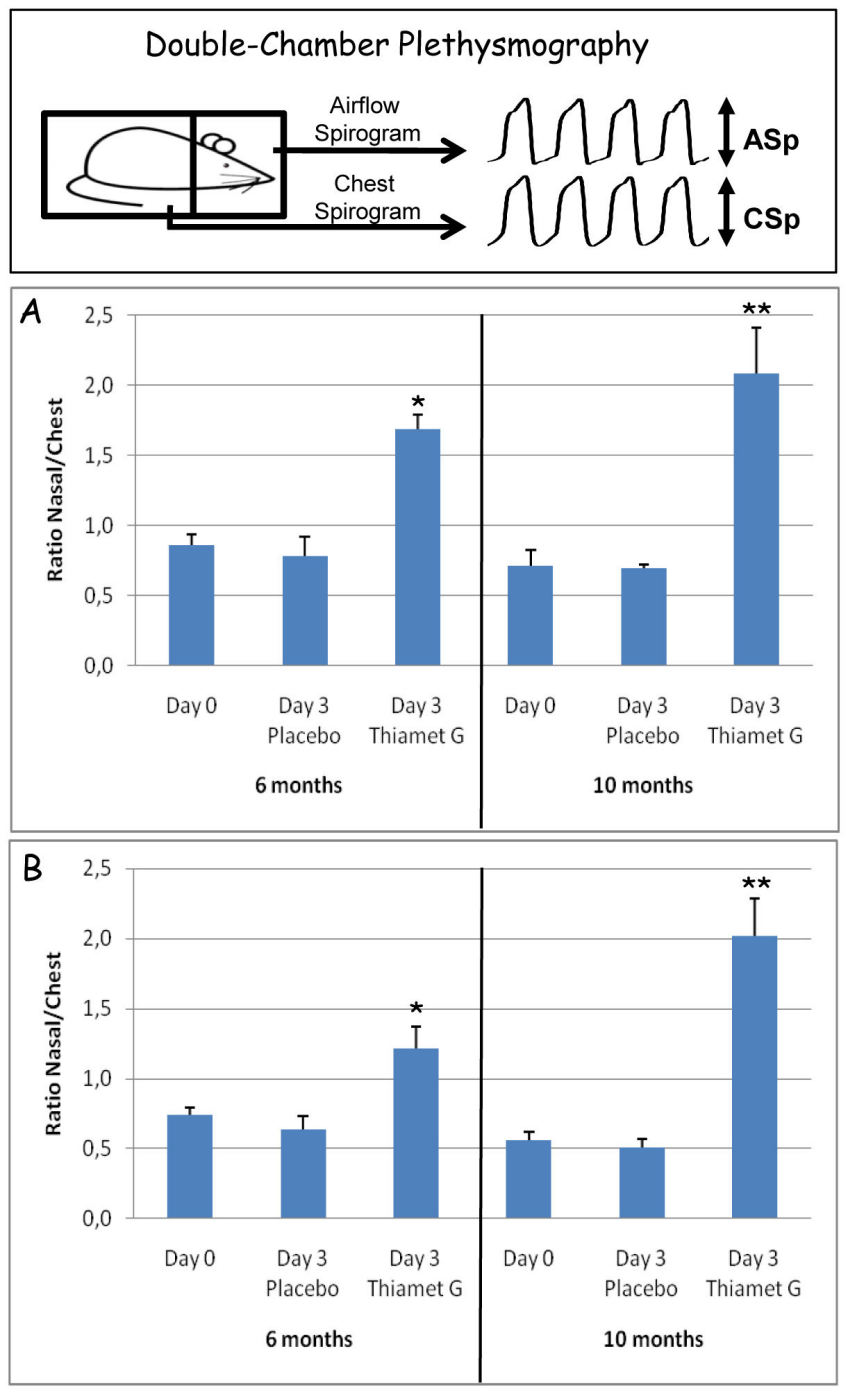
Thiamet-G rescued upper-airway breathing defects of Tau.P301L mice. Dual-chamber plethysmography, schematically represented in the upper panel, was performed as described [25,26]. Treatment of Tau.P301L mice for 3 days with Thiamet-G in the drinking water (2.5 mg/ml) in the home-cage, significantly improved their breathing deficit in normocapnia (panel A) and in hypercapnia (panel B). Of note, the improved breathing efficacy was nearly normalized to that of age-matched wild-type mice (see text for details). Also note that the positive effect was most significant for older Tau.P301L mice. Statistical analysis by ANOVA: *p<0.05, **p<0.01.

Two cohorts of female Tau.P301L mice, respectively aged 6-7 and 9-10 months (n=12 each) were analyzed by double chamber plethysmography to define baseline parameters under normocapnia (day 0, Figure 2A) and hypercapnia (day 0, Figure 2B) as for the wild-type mice. Subsequently, mice were randomized in 4 groups (n=6 per age) with access to drinking water with or without Thiamet-G (2.5 mg/ml). After 3 days, the analysis by double chamber plethysmography was repeated. 

The ratios of airflow/chest amplitudes were at baseline lower than unity in all Tau.P301L mice in normocapnia (Figure 2A) and in hypercapnia (Figure 2B) as reported [25,26]. These ratios remained low in placebo treated Tau.P301L mice, but significantly increased to near-normal levels after 3 days of Thiamet-G administration in both normocapnic and hypercapnic conditions. Of note, the most significant improvement was evident for the older Tau.P301L mice (Figure 2A,B; right panels). 

We concluded that inhibition of OGA significantly increased O-GlcNAc-ylation of brain proteins in vivo and corrected the upper-airway breathing defect of Tau.P301L mice to near-normal values. 

### Chronic OGA inhibition improved clinical condition of ageing moribund Tau.P301L mice

The important normalization of the breathing parameters encouraged us to perform a well-defined chronic treatment study with a high dose of Thiamet-G in Tau.P301L mice, with combined clinical, pathological and biochemical parameters and read-outs. This larger study was additionally promoted by a preliminary 2.5 month treatment of Tau.P301L mice (age 7-9.5 months; n=19) that indicated improved motor-functions and longer survival, without appreciable side-effects.

The chronic study was organized with a cohort of 26 female Tau.P301L mice, specifically bred to yield two homogeneous age-groups for placebo and Thiamet-G treatment (at the start respectively 7.23±0.26 and 7.18±0.28 months, n=13 each). Daily observation and recording of general condition, body-weight and clasping score, was complemented with 2-weekly accelerating rotarod tests. The study was scheduled to cover the age-window of 7 to 9.5 months, respectively marking the onset of motor defects and the median survival age of our Tau.P301L mice (Figure S1). 

Body-weight and clasping were selected as reliable indicators of the late-stage, pre-terminal condition of Tau.P301L mice [13,22-26]. For ethical reasons, we euthanized Tau.P301L mice when 'terminal', which was operationally defined by clasping score 4 (all legs) and bodyweight below 16 grams for female Tau.P301L mice. This condition, denoted 'clasping 5' in some figures, was found to be a reliable indicator of imminent death within 1-3 days in our previous studies [22-26]. Tau.P301L mice that became terminal during the treatment period (placebo n=10/13, Thiamet-G n=5/13) were euthanized by standard methods and brain hemispheres were frozen for biochemical and fixed for immunohistochemical analysis. At age 9.5 months, which was the pre-determined endpoint of the 2.5 month treatment, all surviving Tau.P301L mice (placebo n=3/13, Thiamet-G n=8/13) were euthanized and their brain processed for analysis, identically to the mice that became terminal during the course of the study. All brains were processed and analyzed together.

**Figure 3:**
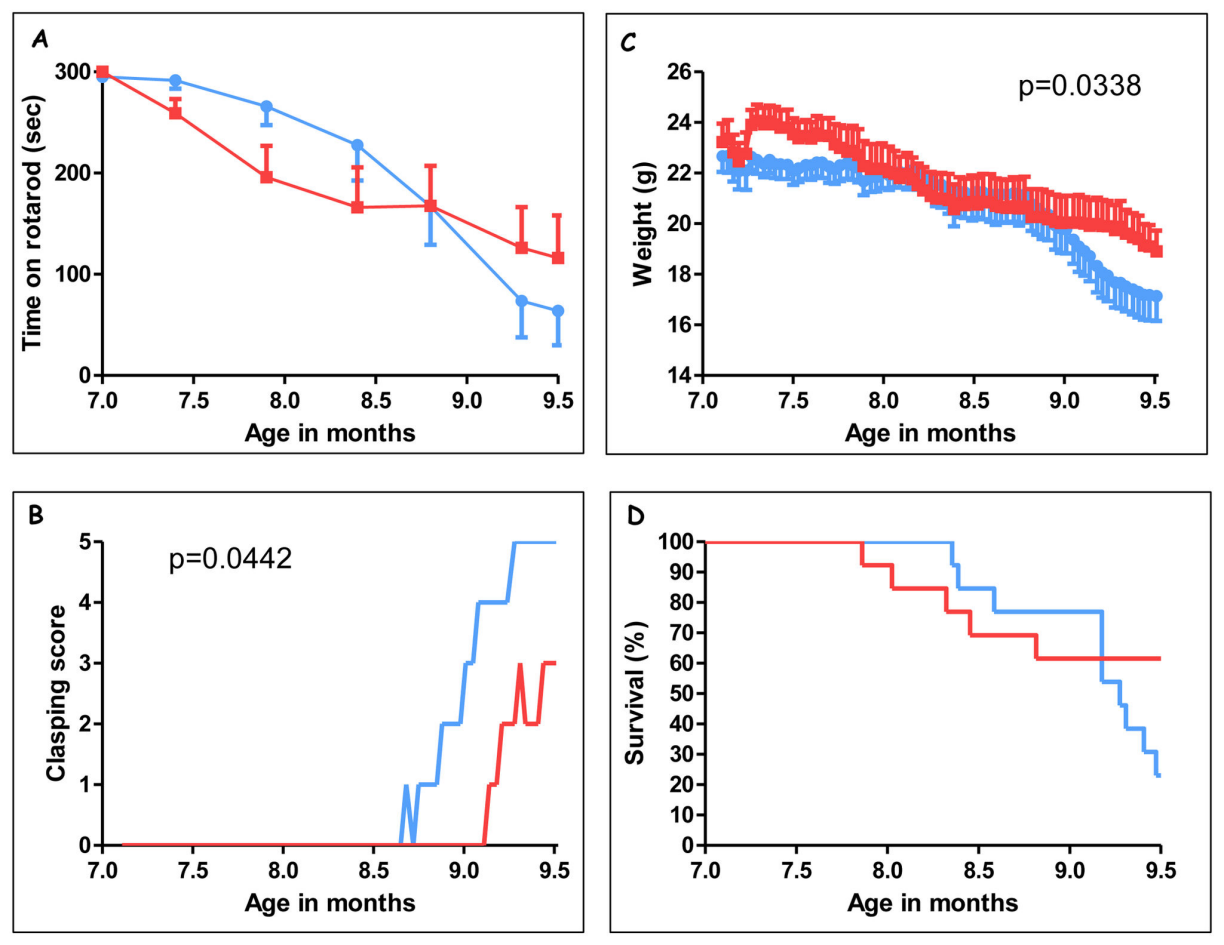
Chronic treatment with Thiamet-G improved clinical parameters of Tau.P301L mice. Female Tau.P301L mice (n=13, age 7.2 months at start) were given access to normal drinking water without (placebo, blue symbols and lines ) or with Thiamet-G (2.5 mg/ml, red symbols and lines) for a total duration of 2.5 months. Mice were monitored by automated accelerating rotarod every 2 weeks (panel A) and daily for clasping (panel B) and body-weight (panel C). Panel D: Kaplan-Meier curves of the two cohorts of Tau.P301L mice (n=13 each). At the predefined endpoint of the study (2.5 months treatment) surviving Tau.P301L mice (n=3/13 for placebo, n=8/13 for Thiamet-G) were sacrificed (see text for details). The endpoint was defined at the start of the experiment to coincide with the median survival age (50% mortality) of Tau.P301L mice in our breeding colony (Fig S2; see text for details) [22-26].

Chronic treatment with Thiamet-G was beneficial for their clinical condition by all parameters: (i) improved motor functions by clasping and in rotarod (Figure 3AB); (ii) alleviated reduction in body-weight (Figure 3C); (iii) delayed precocious death of ageing Tau.P301L mice (Figure 3D). Of note, at the pre-defined endpoint (median survival age of 9.5 months) nearly 3-fold more (n=8/13, 61%) Thiamet-G treated mice were alive, and in far better clinical condition than placebo-treated Tau.P301L mice (n=3/13; 23%) (Figure 3D). 

We concluded that the evolution of the motor functions, assessed by rotarod and by clasping (Figure 3A,B), of body-weight (Figure 3C) and of survival (Figure 3D, compare to overall Kaplan-Meier curve, Figure S1) showed the same beneficial trends and final outcome by treatment with Thiamet-G. Moreover, all clinical parameters correlated tightly with each other and with precocious mortality of ageing Tau.P301L mice (Figure S2). The data further validate this pre-clinical model for tauopathy with respect to these important clinical parameters.

### Immunohistochemical and biochemical analysis of brain after OGA inhibition

**Figure 4:**
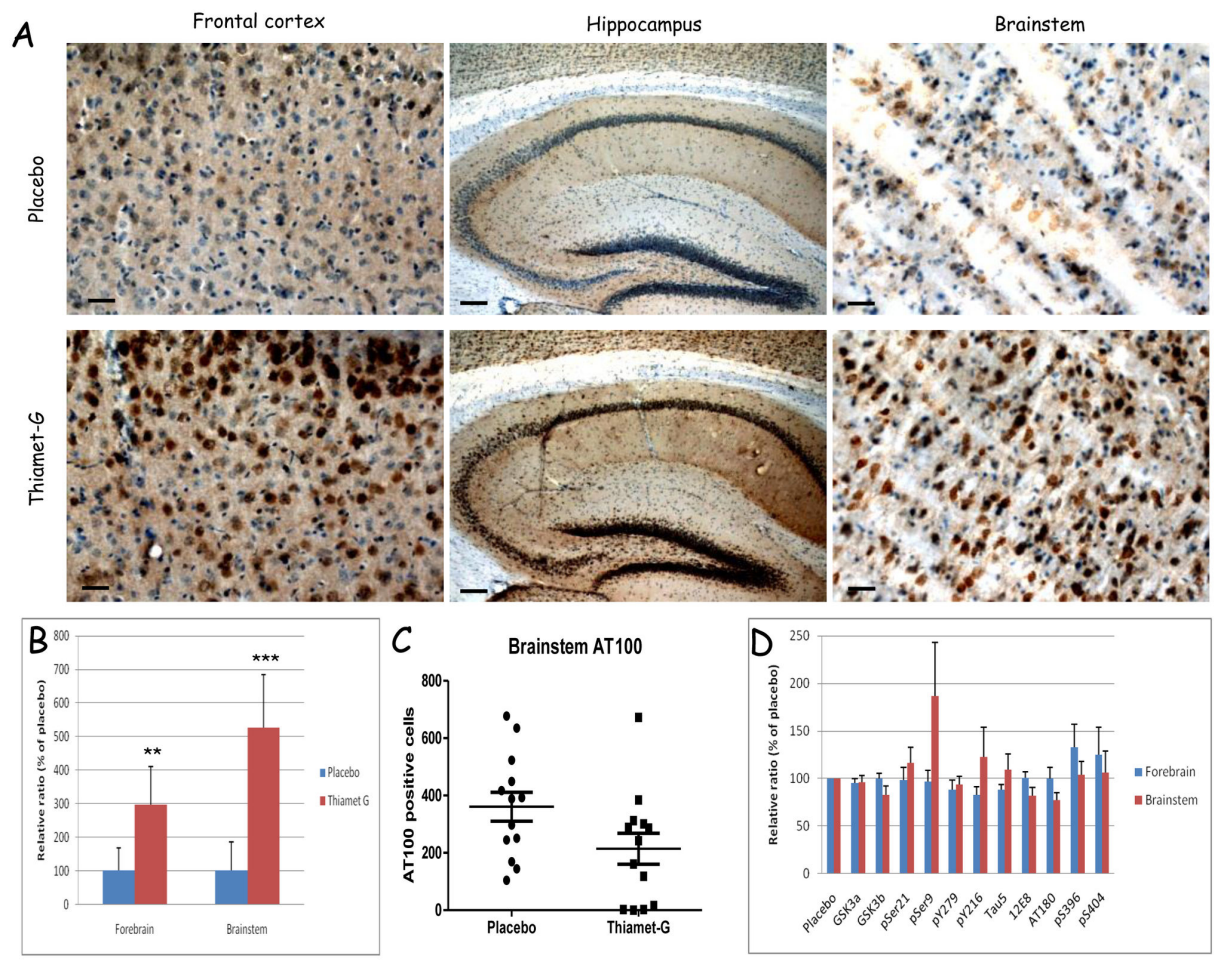
Immunohistochemistry and biochemistry of Tau.P301L mice chronically treated with Thiamet-G. A. Immunohistochemistry (antibody CTD110.6) demonstrated increased O-GlcNAc-ylation in brain regions specified in the captions of Thiamet-G treated Tau.P301L mice (lower panels) versus placebo treated Tau.P301L mice (upper panels). Scalebars: 100 µm and 200 µm in middle panels. B. Quantification of western blotting with CTD110.6 antibody of total protein extracts from forebrain and brainstem of placebo and Thiamet-G treated Tau.P301L mice (** p<0.01,*** p<0.001). C. Quantification of AT100 positive neurons in brainstem of placebo and Thiamet-G treated Tau.P301L mice demonstrating less affected neurons after treatment with Thiamet-G. D. Quantitative data from western blots of forebrain and brainstem extracts from chronically Thiamet-G treated Tau.P301L mice relative to placebo treated Tau.P301L mice (mean±SEM, n=13 each).

Immunohistochemistry demonstrated the expected overall increase in O-GlcNAc adducts in brain of treated Tau.P301L mice (Figure 4A). Because of the well-known close physiological relation of breathing actions to dedicated brainstem circuits, which are both pathologically affected by tauopathy [25,26], we comparatively analyzed forebrain and brainstem. 

Protein O-GlcNAc-ylation was increased significantly more in brainstem than in forebrain (Figure 4B), which correlated inversely with the number of AT100 positive neurons: this was lower in the brainstem of Thiamet-G treated Tau.P301L mice (Figure 4C).

Biochemically analysis by western blotting demonstrated total levels of human and mouse protein tau not to be markedly affected by chronic OGA inhibition (bars labeled Tau5, Figure 4D). Moreover, similar to the acute experiments (Figure 1B), the phosphorylation of protein tau was only marginally affected and, if anything, in the opposite direction in brainstem than in forebrain (Figure 4D). We nevertheless analyzed biochemical parameters of the major tau-kinase GSK-3 [15]. Neither acute nor chronic OGA inhibition affected total levels of the GSK3a/b kinases in forebrain, while the level of GSK3β tended to be lowered in brainstem. Indication for an even further reduction in kinase activity was testified by the higher inhibitory phosphorylation pSer9-GSK3β (Figure 4D). 

We went on to perform western blotting with commercially available antibodies against O-GlcNAc adducts of brain proteins of untreated and Thiamet-G treated mice, and we also included Tau.KO mice deficient in protein tau (denoted TKO in the figures) [27]. As expected, the antibodies yielded biochemical patterns with important qualitative and quantitative differences (Figure S3). Antibodies CTD110.6, RL2 and 6D93 yielded the most consistent biochemical signals in western blotting (Figure 1, Figure S3) and in immunohistochemistry (Figure 4). As remarked in the acute experiments, also chronic treatment with Thiamet-G failed to reveal O-GlcNAc-labeled proteins in the brain extracts that could correspond directly in electrophoretic mobility to human Tau.P301L (64-68 kDa) or to mouse protein tau (~55kDa) (Figure S3). 

Moreover, these and other biochemical data (described below) demonstrated that the in vivo effect of Thiamet-G on brain proteins was largely, if not exclusively, quantitative in nature: an increase in extent of O-GlcNAc-ylation, without a marked increase in the number of O-GlcNAc-ylated brain proteins. 

### Protein Tau.P301L is not O-GlcNAc-ylated in brain of Tau.P301L mice

We noted that none of the antibodies highlighted in western blotting an O-GlcNAc-ylated protein that migrated similarly to human Tau.P301L (60-64 kDa) or murine tau (~55 kDa) (Figure 1A; Figure S3, red broken line). Moreover, O-GlcNAc-ylated proteins in brain of Tau.KO mice deficient in protein tau were very similar to those of Tau.P301L mice, either treated or untreated with Thiamet-G (Figure S3, lanes 1 and 2 in all panels). Potentially interesting was the observation of brain proteins in Tau.KO mice that reacted with antibodies 1F5.D6 and 18B10.C7 (Figure S3). 

To circumvent technical inconsistencies, we additionally prepared and analyzed recombinant protein tau that was O-GlcNAc-ylated in vitro by recombinant OGT, to be used as positive control for validating the western blotting procedures as conform published methods. Moreover, we used buffers containing as blocking agent 5% bovine serum albumin (BSA) because it is not glycosylated, essential to obtain clean and strong signals in western blotting with the antibodies against O-GlcNAc-adducts. Several other variables and parameters were implemented and tested: pretreating western blots after transfer with alkaline phosphatase to increase the signal, and the inclusion of Thiamet-G or N-acetyl-glucosamine during homogenization and sample preparation. The latter procedural modifications improved somewhat the signal-to-noise ratio in western blotting but yielded essentially no extra information. 

**Figure 5:**
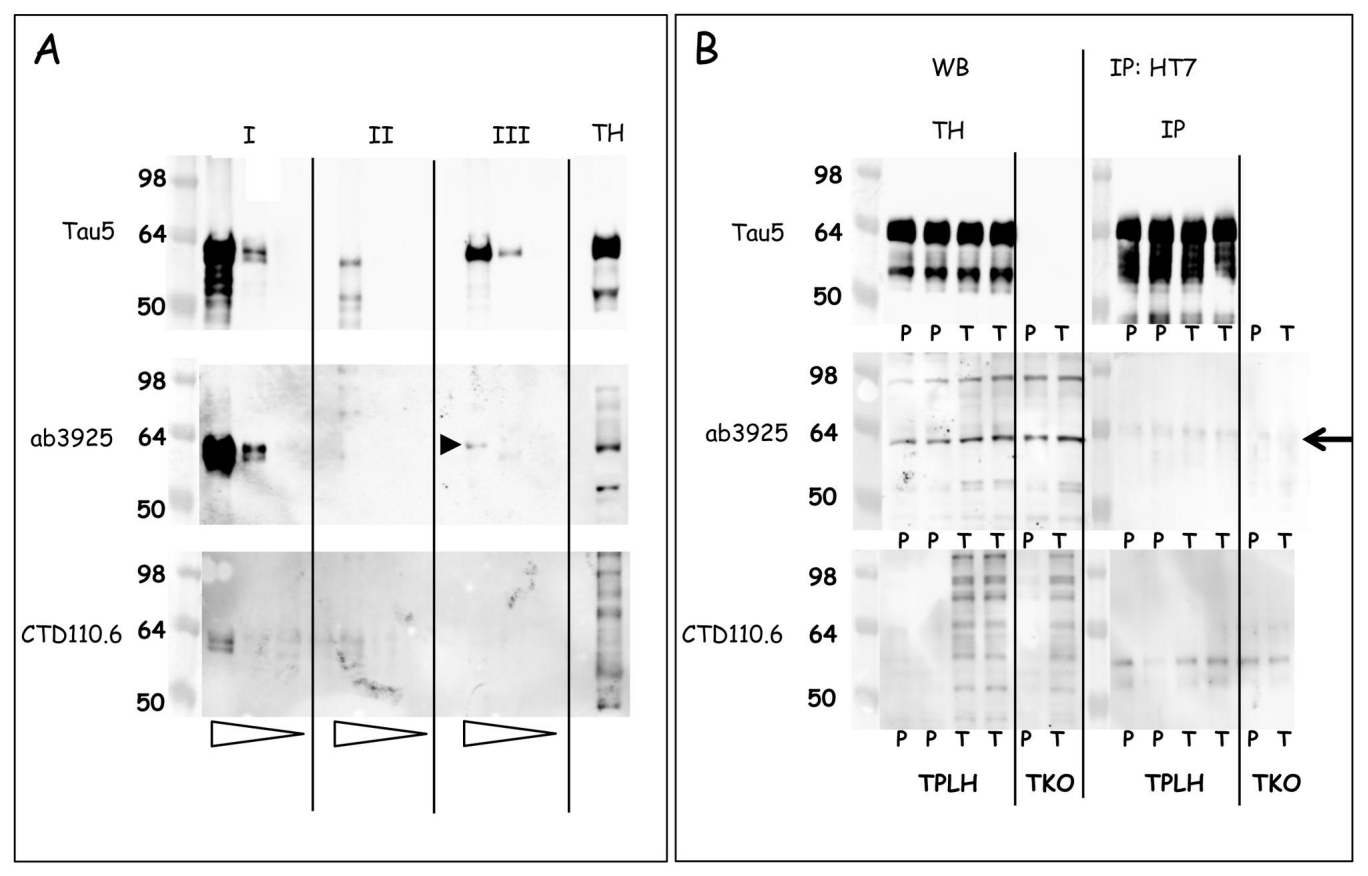
Biochemical analysis demonstrating that protein tau is not O-GlcNAc-ylated in mouse brain. A. Western blotting with antibodies Tau5, ab3925 and CTD110.6 of (I) recombinant Tau O-GlcNAc-ylated in vitro with OGT [21]; (II) human tau and OGT co-expressed in E.Coli [10,11]; (III) recombinant tau441. Samples were loaded in 10-fold serial dilutions indicated by the open triangles underneath the lanes. A sample of total brain extract is included (denoted TH). The black arrowhead denotes the cross-reaction of ab3925 with recombinant protein tau, discussed in the text. B. Immunoprecipitation of protein tau with antibody HT7 from brain extracts of Tau.P301L and from Tau.KO mice, either untreated (lanes marked P) or treated with Thiamet-G (lanes marked T). The arrow denotes the 64 kDa protein that is detected with ab3925 in all mice, including Tau.KO mice, as discussed in the text.

We analyzed three different types of samples (denoted by roman symbols in Figure 5A): samples I contained recombinant protein tau O-GlcNAc-ylated in vitro with recombinant OGT [11,21]; samples II were extracts from bacterial cultures that co-expressed human protein tau and OGT [11,21]; samples III consisted of commercial recombinant protein tau without further treatment. In all panels three amounts of these samples were loaded (serial 10-fold dilutions) next to a total brain protein extract from a Thiamet-G treated Tau.P301L mouse (last lane denoted TH, Figure 5A). 

Western blotting demonstrated antibody CTD110.6 to react with in vitro O-GlcNAc-ylated recombinant protein tau (samples I, lower panel, Figure 5A) but not with protein tau co-expressed with OGT in bacteria (samples II, lower panel, Figure 5A). The latter was attributed to the fact that co-expression with OGT diminished the expression levels of protein tau in the bacteria, as exemplified by western blotting with Tau5 for total protein tau (Figure 5A, upper panel). 

Antibody ab3925 was tested because it was specifically raised against the O-GlcNAc-ylated S400 epitope in protein tau [10,11]. The antibody ab3925 reacted more strongly with samples I, but again not with protein tau expressed in bacteria, although some high Mr bacterial proteins cross-reacted (samples II, middle panel, Figure 5A). Unexpectedly, ab3925 cross-reacted with the commercial recombinant protein tau that was otherwise untreated (samples III, middle panel, Figure 5A; black arrowhead). This cross-reaction of ab3925 with recombinant protein tau was not observed in western blotting with CTD110.6 on the same samples in otherwise identical conditions (samples III, lower panel, Figure 5A). In addition, ab3925 reacted with several proteins in the total brain protein extract from a Thiamet-G treated Tau.P301L mouse (lane denoted TH, middle panel, Figure 5A). 

We went on to analyze protein tau in total brain extracts of Tau.P301L mice, both untreated (lanes denoted P for placebo, Figure 5B) and treated with Thiamet-G (lanes denoted T, Figure 5B). We used the same antibodies in direct western blotting (left panels, Figure 5B) and after immunoprecipitation of Tau.P301L with antibody HT7, specific for human tau (right panels, Figure 5B). In addition we analyzed brain protein extracts from mice deficient in protein tau (denoted TKO, Figure 5B), treated or not with Thiamet-G (respectively denoted T or P) (Figure 5B). 

Direct western blotting (denoted WB, left panels, Figure 5B) yielded the expected pattern of total human and mouse tau with antibody Tau5, unchanged by treatment with Thiamet-G and lacking completely in Tau.KO mice (upper panel, Figure 5B). The pattern obtained for O-GlcNAc-ylated proteins with antibody CTD110.6 was also as expected, including the marked increase by treatment with Thiamet-G (compare lanes denoted T to lanes denoted P, lower panel, Figure 5B). Note the very similar pattern observed in the brain of Thiamet-G treated Tau.KO mice that are completely deficient in protein tau (lower panel, lanes denoted TKO, Figure 5B). 

In contrast to antibody CTD110.6, the antibody ab3925 failed markedly to distinguish between treated and untreated Tau.P301L mice. Moreover, ab3925 even yielded a very similar brain protein pattern from Tau.KO mice as for Tau.P301L mice, while not markedly different for either untreated or treated with Thiamet-G (middle panel, Figure 5B). 

Surprisingly, a distinct protein with apparent Mr of approximately 64 kDa, comparable to that of non-phosphorylated human protein tau, was revealed with ab3925 in all brain extracts from Tau.P301L mice and also from Tau.KO mice, independent of treatment with Thiamet-G (middle panels, Figure 5B; Figure S4). Obviously, besides cross-reaction with recombinant protein tau demonstrated in the previous section, antibody ab3925 cross-reacted with several other murine brain proteins unrelated to protein tau, including one with apparent Mr 64 kDa (large arrow, middle panel, Figure 5B). Moreover, analysis of more mice revealed that this 64 kDa protein became somewhat more O-GlcNAc-ylated in brain of Tau.P301L mice treated with Thiamet-G (second panel, Figure S4). Further analysis on more samples of untreated and treated Tau.P301L and Tau.KO mice confirmed these data (Figure S5). 

We went on to perform immunoprecipitation of protein tau with antibody HT7, specific for human protein tau, followed by western blotting with the same three antibodies: Tau5, ab3925 and CTD110.6. This approach failed to reveal marked differences in brain proteins between untreated (samples denoted P) and Thiamet-G treated (samples denoted T) Tau.P301L mice (panels on the right, Figure 5B). Western blotting with several other antibodies confirmed this outcome. Moreover, the 64 kDa protein that was visualized by direct western blotting with ab3925 was not observed after specific immunoprecipitation with HT7, confirming that it was unrelated to protein tau (large arrow, middle panel, Figure 5B; see also Figure S4, S5). 

Our combined biochemical data demonstrated that protein tau was not O-GlcNAc-ylated in mouse brain, neither at baseline nor after treatment of the mice with Thiamet-G. We concluded that Thiamet-G rapidly increased O-GlcNAc-ylation of brain proteins in Tau.P301L mice, while protein tau and its phosphorylated isoforms were not markedly affected. 

## Discussion

Our data provide independent pre-clinical evidence that a CNS-related disorder was beneficially affected by pharmacologically increasing protein O-GlcNAc-ylation. In a validated pre-clinical model of tauopathy, the designed OGA-inhibitor Thiamet-G [10,11] increased the O-GlcNAc-ylation of many proteins rapidly (hours) and stably (months) in wild-type and transgenic mouse brain. The pharmacological effectiveness included mitigation of reduced body-weight and improved motor abilities, indirectly corroborating the motor defects of OGT-deficient mice [6,7]. Moreover, three-fold more Tau.P301L mice survived, in close concert with their normalized upper-airway breathing capacity that we demonstrated to originate in the tau-laden brainstem nuclei and circuits that control breathing actions [25,26]. 

The Tau.P301L mice were treated with Thiamet-G by adding the compound to the drinking water at a concentration of 2.5 mg/ml. Based on normal water consumption of 5 ml/day, the dose would amount to about 500 mg/kg bodyweight. Despite this rather high dose, the overall outcome of all our acute, sub-acute and chronic studies proved that the compound not only effectively exerted therapeutic effects, but equally important that inhibition of OGA can be considered innocuous in vivo. Moreover, we performed a follow-up study in the same mouse model with the same compound added to the drinking water at only 1 mg/ml, yielding qualitatively similar improvements of the four clinical parameters, although quantitatively less marked (Figure S6). Obviously, these findings should and will further promote the search for novel compounds as more efficacious inhibitors of OGA that enter the brain more readily or even preferentially [28]. 

Unexpectedly, Thiamet-G affected the phosphorylation of protein tau in vivo only marginally and at a limited set of epitopes. Interesting in this respect were the changes in opposite directions in brainstem versus forebrain, leading to significant lowering of the number of brainstem neurons containing tauopathy that we have defined previously by IHC with antibody AT100 [22-26]. The marked reduction in neurons containing tau aggregates in the brainstem by OGA inhibition thereby corroborated the significant mitigation of upper airway defects by Thiamet-G, demonstrated here. Nevertheless, in depth analysis of the specific neuronal circuits that control breathing will be needed to establish the exact relation. Conversely, we believe that the pathological and clinical implications are important, although the brainstem is neglected in post-mortem pathological analysis of patients with tauopathies and AD. 

The neuron-specific expression of human mutant Tau.P301L is the evident direct cause of the functional defects and of the neuronal pathology in this pre-clinical transgenic model. The observed beneficial effects must then be attributed to increased O-GlcNAc-ylation of neuronal proteins, because our data explicitly exclude protein tau itself. Other selected candidates, e.g. GSK3α/β and its upstream controller Akt/PKB, as well as PP2A, among other brain proteins surmised to be O-GlcNAc-ylated [29-31] were not supported as direct targets by our biochemical analysis (Figure S7). The responsible targets that become O-GlcNAc-ylated and counteract the negative effects of Tau.P301L, must then be sought downstream in the pathological cascade initiated by the mutant protein tau. 

Our data illustrated once more the complex relation of phosphorylation, aggregation into AT100-positive fibrils and functional neurotoxicity in tauopathy, including AD [12-15]. Of note, the intra- and extra-neuronal whereabouts of protein tau are not only controlled by kinases and phosphatases, but also by chaperones and heat-chock proteins, several of which are known clients of O-GlcNAc-ylation [1,5,30]. In addition, O-GlcNAc-ylated neuronal proteins participate in synaptic actions underlying learning and memory, and promoting long-term potentiation [32]. Obviously, the brainstem circuits that control the complex action of breathing have acquired this information by learning and store these involuntary actions in memory, relying on specialized synaptic contacts. 

The overall outcome of our efforts was unexpected but welcome, both in terms of the interesting clinical effects on the Tau.P301L mice in vivo, as well as on the absence of O-GlcNAc-ylation of protein tau itself. Not only the former, but also the latter is regarded as an asset, because the identification of primary protein-clients for OGA in brain will eventually define the actions of O-GlcNAc-ylation in pathology and physiology. Moreover, molecular details on how wild-type or mutant protein tau inflicts the clinical phenotype in a specific tauopathy, will provide an interesting outlook on novel therapeutic targets for neurodegenerative diseases, caused or mediated in first or second order by protein tau. 

Conversely, our current data remain consistent with the hypothesis that O-GlcNAc-ylation is involved in neurological diseases, including AD [8,9], although with the important caveat that protein tau itself is not directly modified. Unfortunately, in most studies, O-GlcNAc-ylation of protein tau was studied and demonstrated only on recombinant tau or in overexpressing cell-models, and not in brain in vivo. 

Obviously, O-GlcNAc-ylation of protein tau continues to be the subject of technical problems and controversy. To our knowledge, the evidence that protein tau is O-GlcNAc-ylated in brain in vivo has been circumstantial and not conclusive because of technical or procedural issues. In this study, we are the first to unequivocally claim that O-GlcNAc-ylation of protein tau in mouse brain was not detectable by the extensive combination of biochemical methods presented here. 

Finally, the obvious minor or even absent side-effects in our three long-term studies is evidently an important asset and urge for the development of more efficacious inhibitors of OGA, eventually targeted more directly to the CNS [28]. The general increase of O-GlcNAc-ylation of proteins is here demonstrated to be a well-tolerated post-translational modification, corroborating the severe defects caused by lacking or decreased O-GlcNAc-ylation [1-7]. Our results open interesting prospects for treating patients suffering from tauopathies, and possibly other diseases caused by aggregated proteins in debilitating CNS disorders. 

## Supporting Information

Figure S1 **Kaplan-Meier mortality curves of male and female Tau.P301L mice.** Mortality data are collected over 6 years in our breeding colony of Tau.P301L mice and the data-set concerns 411 male and 1206 female Tau.P301L mice. Despite some initial bias towards females, the 50% median survival age is nearly identical (~9 months) for both genders. As stated before, practically no Tau.P301L mice survive for more than 12 months [22-26].
(TIF)

Figure S2 **Correlation of the clinical parameters of Tau.P301L mice.** All available data-sets of rotarod, clasping, body-weight and survival (relative percentage) of untreated and Thiamet-G treated female Tau.P301L mice were correlated in the six possible combinations. The correlation coefficients (R^2^) indicated in the graphs, ranged from 0.8004 to 0.9483 for the survival-rotarod and the clasping-rotarod paired correlations, respectively.
(TIF)

Figure S3 **Comparative western blots with commercial antibodies against O-GlcNAc protein adducts.** Western blots with available antibodies against O-GlcNAc (Table 1) of total forebrain extracts from Tau.KO mice (lanes 1,2) and from Tau.P301L mice (lanes 3-6), either untreated (lanes 1, 3, 4) or Thiamet-G treated (lanes 2, 5, 6; bold in the captions above the blots).
The red broken line delineates the electrophoretic mobility of protein Tau.P301L.
(TIF)

Figure S4 **Western with ab3925 for O-GlcNAc-S400 compared to total O-GlcNAc in Tau.P301L mice.** Brain extracts from Tau.P301L mice, untreated (placebo) or treated with Thiamet-G (drinking water 2.5 mg/ml for 3 days) were analyzed by western blotting. The red boxes denote the proteins quantified (lower panels). Note the significant increased O-GlcNAc-ylation revealed with antibodies RL2 and CTD110.6 in contrast to the non-significant increased 64 kDa protein detected with ab3925. The arrowhead denotes an extra protein detected by ab3925 and CTD110.6 in treated mice, not further analyzed here. The actin loading control (*) was needed for quantification and superimposed graphically on the blots shown.
(TIF)

Figure S5 **Western blotting with ab3925 and CTD110.6 of Tau.P301L and Tau.KO mice.** Western blots with antibody Tau5 for total protein tau, and with ab3925 and CTD110.6 of total forebrain extracts from Tau.P301L mice (lanes denoted TPLH) and from Tau.KO mice (lanes denoted TKO) either untreated (lanes denoted Plac) or treated with Thiamet-G (lanes denoted TGX). The red rectangle (solid line) delineates the electrophoretic mobility and position of protein Tau.P301L (denoted hTau). The blue rectangle (broken line) denotes the 64 kDa protein detected by ab3925 in all mouse brain extracts, including Tau.KO mice. We further noted cross-reaction of ab3925 with several brain proteins, including strong reaction with a 40 kDa unknown protein in all brain extracts.
(TIF)

Figure S6 **Beneficial effects of Thiamet-G at lower dose in Tau.P301L mice.** Female Tau.P301L mice (age 6-7 months at start) were supplied drinking water without (placebo) (n=8) or with Thiamet-G (1.0 mg/ml) (n=10) for 3.5 months. Mice were monitored weekly by automated accelerating rotarod (panel A), clasping (panel B) and body-weight (panel C).
Panel D: mortality (see Figure 4, Figure S2 and text for details).
(TIF)

Figure S7 **Western blotting for Akt/PKB and PP2A in brain extracts of Tau.P301L mice.** Representative western blots and quantitative analysis of total brain extracts from placebo (P) and Thiamet-G (T) treated Tau.P301L mice (n=13 each) with the specified antibodies (Table 1).
(TIF)
