# Supplementary figures and images for: Increasing Brain Protein O-GlcNAc-ylation Mitigates Breathing Defects and Mortality of Tau.P301L Mice
Source: PLoS One. 2013 Dec 23;8(12):e84442. doi: 10.1371/journal.pone.0084442 (PMC3871570; doi:10.1371/journal.pone.0084442)

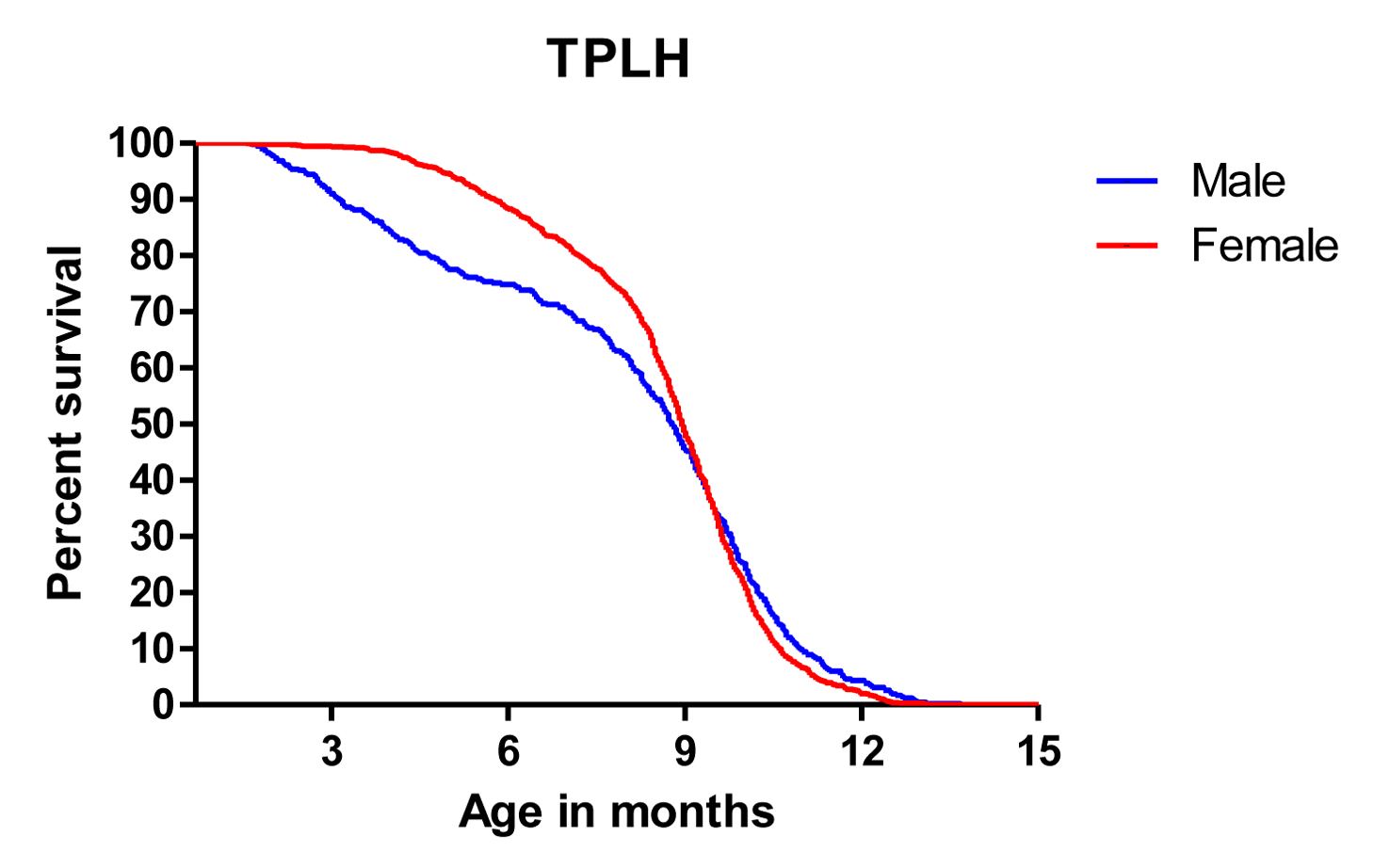

Supplement: Figure S1 — Kaplan-Meier mortality curves of male and female Tau.P301L mice. Mortality data are collected over 6 years in our breeding colony of Tau.P301L mice and the data-set concerns 411 male and 1206 female Tau.P301L mice. Despite some initial bias towards females, the 50% median survival age is nearly identical (~9 months) for both genders. As stated before, practically no Tau.P301L mice survive for more than 12 months [22-26]. (TIF) [file pone.0084442.s001.tif]

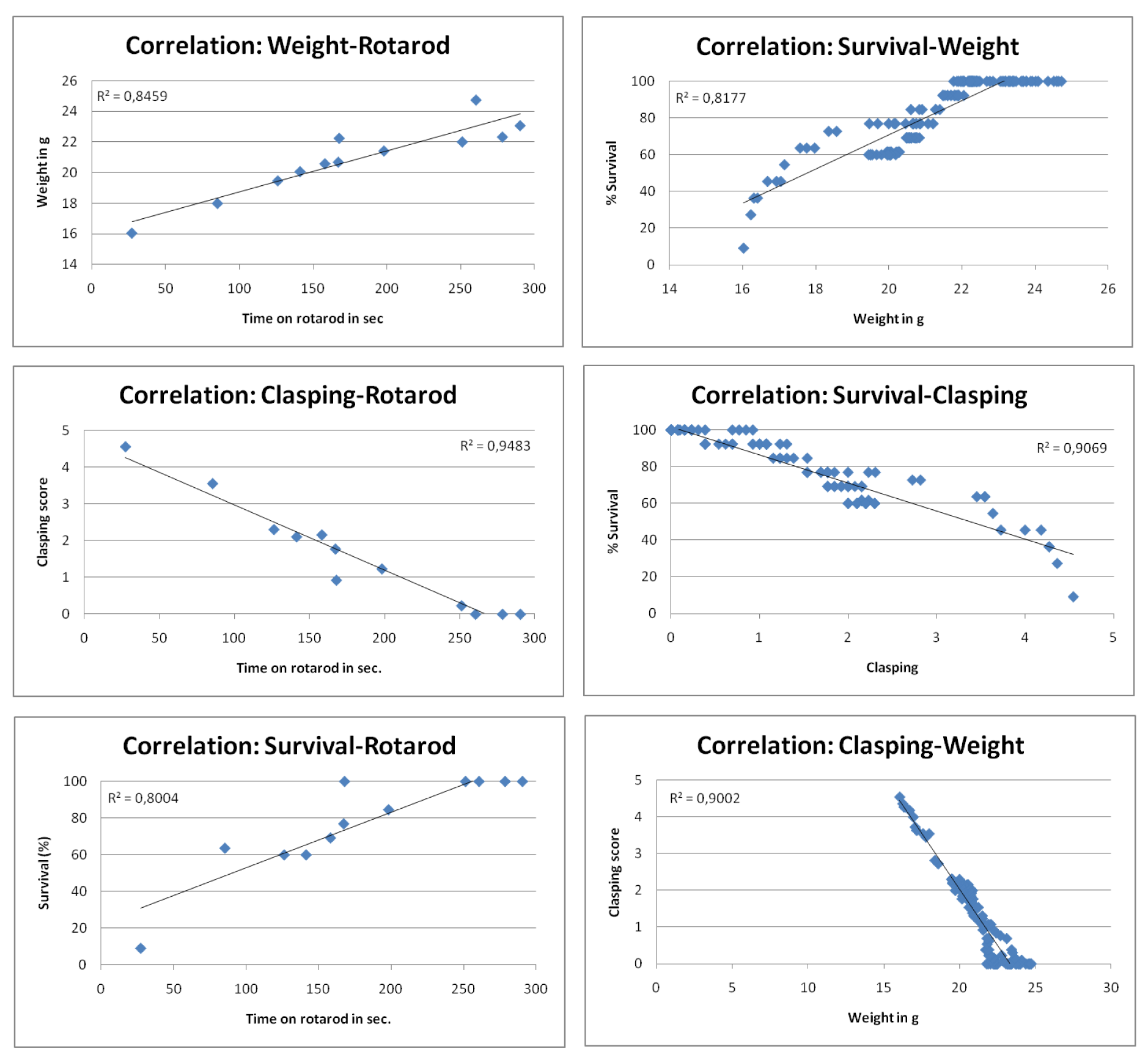

Supplement: Figure S2 — Correlation of the clinical parameters of Tau.P301L mice. All available data-sets of rotarod, clasping, body-weight and survival (relative percentage) of untreated and Thiamet-G treated female Tau.P301L mice were correlated in the six possible combinations. The correlation coefficients (R2) indicated in the graphs, ranged from 0.8004 to 0.9483 for the survival-rotarod and the clasping-rotarod paired correlations, respectively. (TIF) [file pone.0084442.s002.tif]

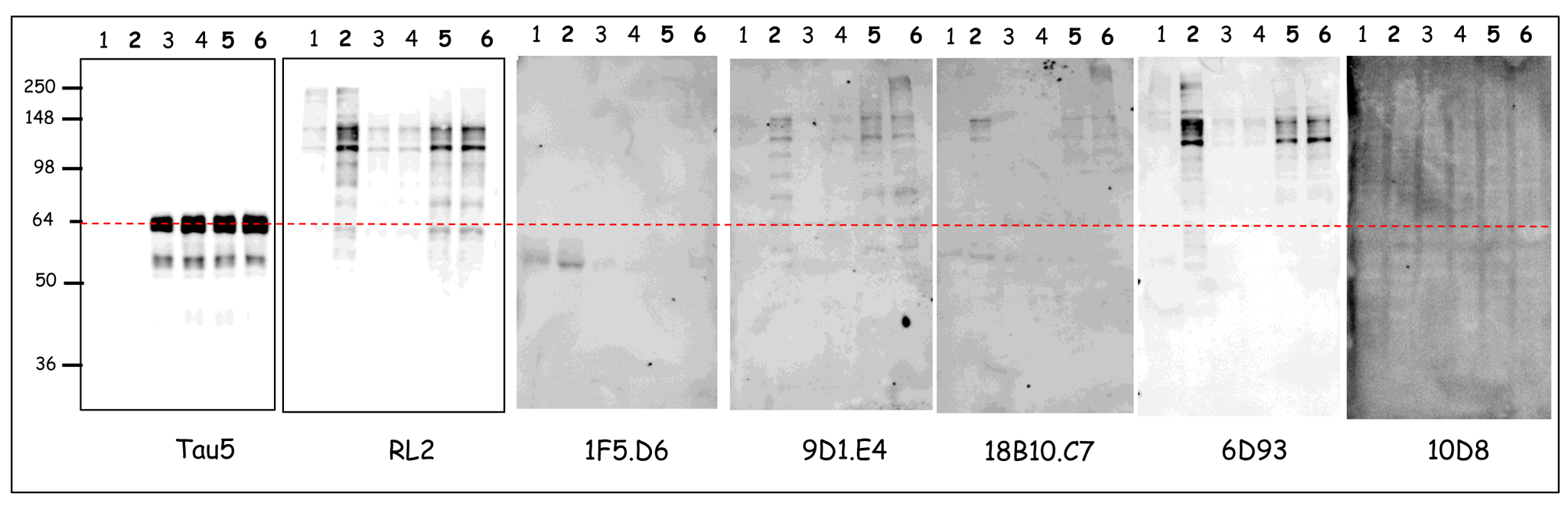

Supplement: Figure S3 — Comparative western blots with commercial antibodies against O-GlcNAc protein adducts. Western blots with available antibodies against O-GlcNAc (Table 1) of total forebrain extracts from Tau.KO mice (lanes 1,2) and from Tau.P301L mice (lanes 3-6), either untreated (lanes 1, 3, 4) or Thiamet-G treated (lanes 2, 5, 6; bold in the captions above the blots). The red broken line delineates the electrophoretic mobility of protein Tau.P301L. (TIF) [file pone.0084442.s003.tif]

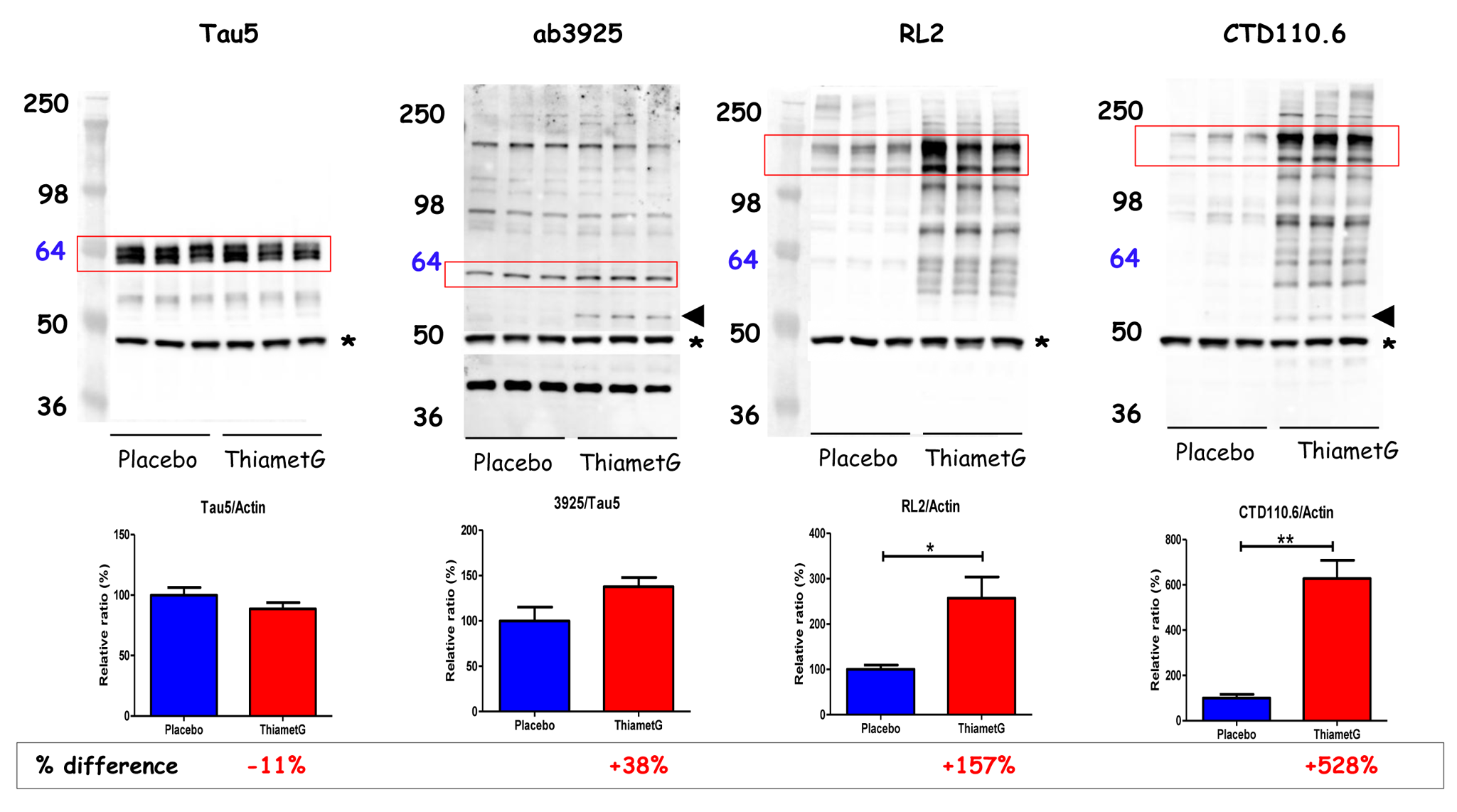

Supplement: Figure S4 — Western with ab3925 for O-GlcNAc-S400 compared to total O-GlcNAc in Tau.P301L mice. Brain extracts from Tau.P301L mice, untreated (placebo) or treated with Thiamet-G (drinking water 2.5 mg/ml for 3 days) were analyzed by western blotting. The red boxes denote the proteins quantified (lower panels). Note the significant increased O-GlcNAc-ylation revealed with antibodies RL2 and CTD110.6 in contrast to the non-significant increased 64 kDa protein detected with ab3925. The arrowhead denotes an extra protein detected by ab3925 and CTD110.6 in treated mice, not further analyzed here. The actin loading control (*) was needed for quantification and superimposed graphically on the blots shown. (TIF) [file pone.0084442.s004.tif]

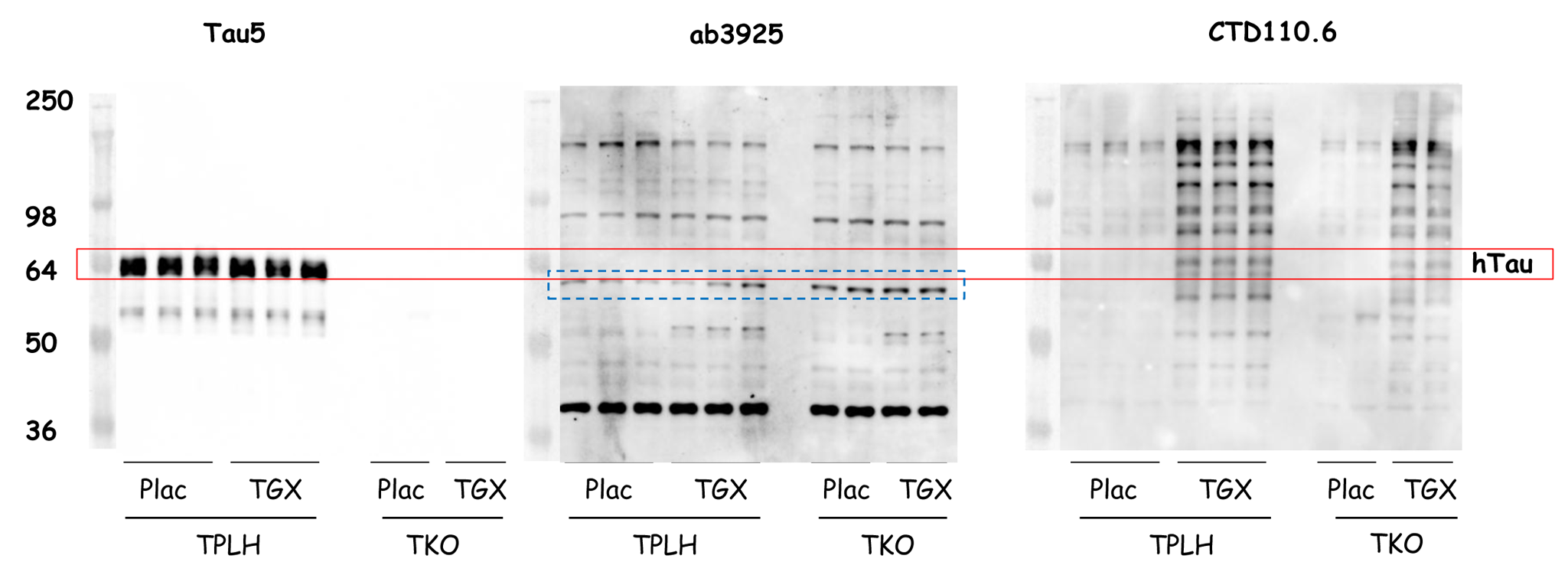

Supplement: Figure S5 — Western blotting with ab3925 and CTD110.6 of Tau.P301L and Tau.KO mice. Western blots with antibody Tau5 for total protein tau, and with ab3925 and CTD110.6 of total forebrain extracts from Tau.P301L mice (lanes denoted TPLH) and from Tau.KO mice (lanes denoted TKO) either untreated (lanes denoted Plac) or treated with Thiamet-G (lanes denoted TGX). The red rectangle (solid line) delineates the electrophoretic mobility and position of protein Tau.P301L (denoted hTau). The blue rectangle (broken line) denotes the 64 kDa protein detected by ab3925 in all mouse brain extracts, including Tau.KO mice. We further noted cross-reaction of ab3925 with several brain proteins, including strong reaction with a 40 kDa unknown protein in all brain extracts. (TIF) [file pone.0084442.s005.tif]

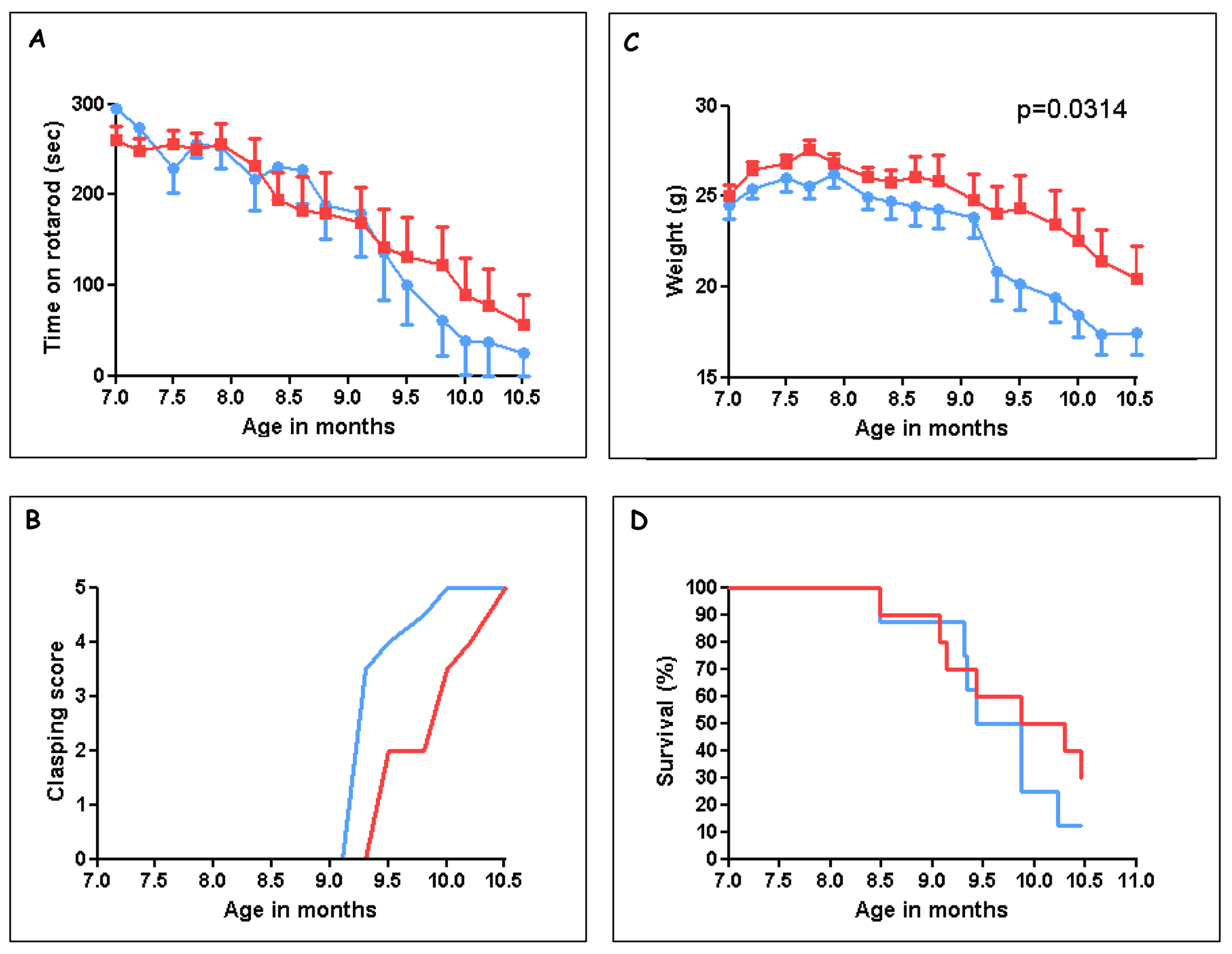

Supplement: Figure S6 — Beneficial effects of Thiamet-G at lower dose in Tau.P301L mice. Female Tau.P301L mice (age 6-7 months at start) were supplied drinking water without (placebo) (n=8) or with Thiamet-G (1.0 mg/ml) (n=10) for 3.5 months. Mice were monitored weekly by automated accelerating rotarod (panel A), clasping (panel B) and body-weight (panel C). Panel D: mortality (see Figure 4, Figure S2 and text for details). (TIF) [file pone.0084442.s006.tif]

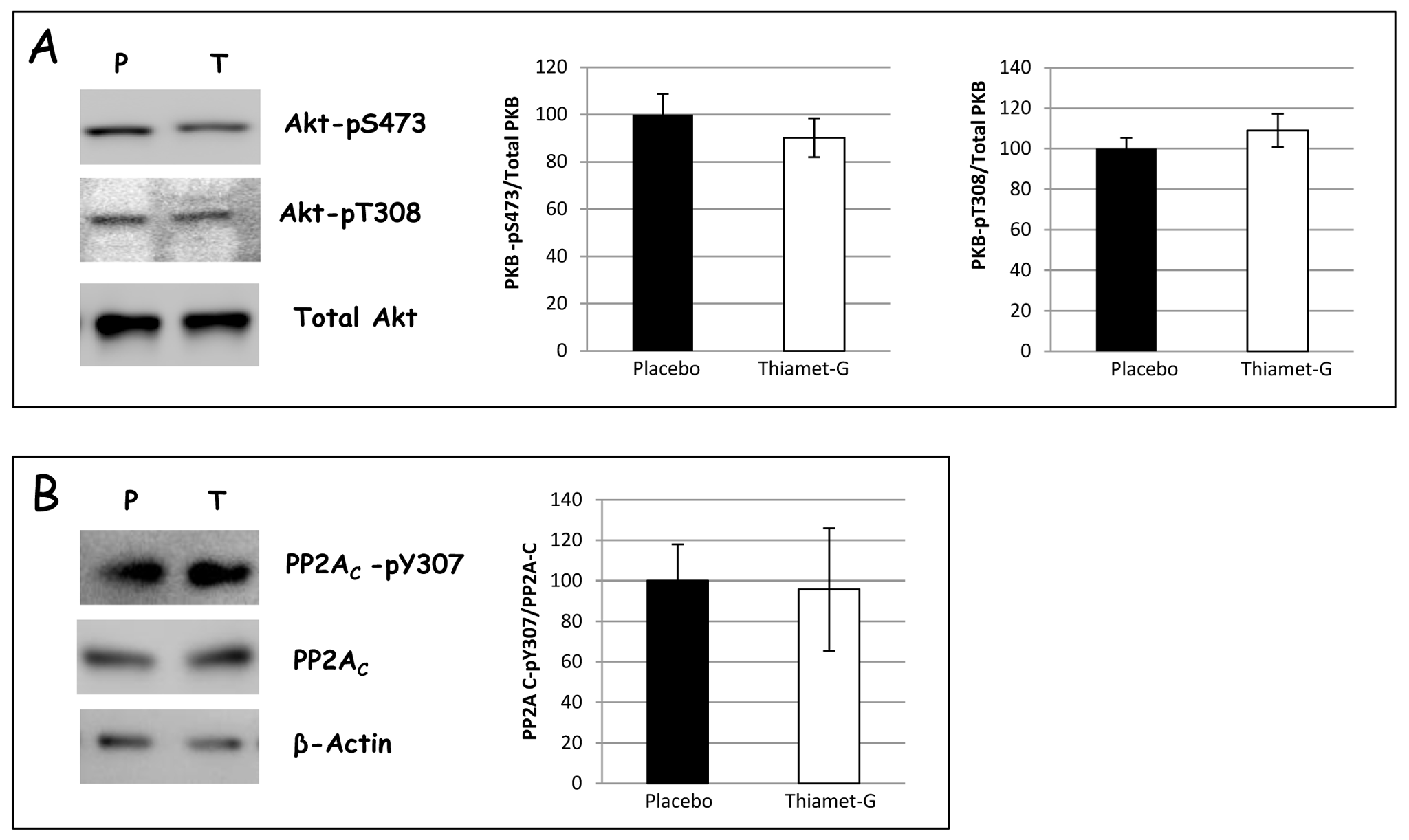

Supplement: Figure S7 — Western blotting for Akt/PKB and PP2A in brain extracts of Tau.P301L mice. Representative western blots and quantitative analysis of total brain extracts from placebo (P) and Thiamet-G (T) treated Tau.P301L mice (n=13 each) with the specified antibodies (Table 1). (TIF) [file pone.0084442.s007.tif]
